# P-466. Predictors of Adverse Clinical Outcomes in Children with Community-Acquired Bacterial Meningitis

**DOI:** 10.1093/ofid/ofaf695.681

**Published:** 2026-01-11

**Authors:** Yara Neaimeh, Elizabeth A Aguilera, Henry Welch, Lydia A Barakat, Christiane Hadi, Gloria P Heresi, Rodrigo Hasbun

**Affiliations:** McGovern Medical School at UTHealth, Houston, Houston, TX; UTHealth, McGovern Medical School, Houston, Texas; Baylor College of Medicine, Houston, Texas; Yale School of Medicine, New Haven, Connecticut; UPMC, Pittsburgh, Pennsylvania; University of texas Health and science at Houston, Houston, Texas; UT Health Mc Govern Medical School, Houston, Texas

## Abstract

**Background:**

Community-acquired bacterial meningitis (CABM) in infants and children is a neurological emergency, requiring immediate evaluation and management. Despite timely management, children with bacterial meningitis remain at risk for adverse clinical outcomes (ACO). The aim of the study was to identify predictors of ACO (i.e., neurologic sequelae, hearing loss and mortality) in children with CABM.

**Methods:**

We conducted a retrospective, multicenter study of three pediatric cohorts (age> 2 months-17 years) with the diagnosis of CABM. Cohort 1 consisted of 195 children from Yale New Haven Hospital from 1/1/1985-2/1/1998. Cohort 2 included 166 children from two New Orleans hospitals (Charity Hospital and Children’s Hospital of New Orleans) from 2/1/1980-4/1/2005, and cohort 3 consisted of 28 children in Houston, Texas between October 2017 and December 2023. Children with ventriculoperitoneal shunt infections were excluded. Baseline patient characteristics, clinical presentation, diagnostic evaluations, treatment and outcomes were evaluated.

**Results:**

A total of 389 patients were included. Of these, 221 (57%) were male. The majority were African American (65.8%) followed by White patients (26.2%). 53 patients (14.9%) had a history of prematurity, 18 patients (5.1%) had developmental delays, and 11 (2.8%) were immunocompromised. ACO were observed in 168 children (44%); 75 (33%) developed hearing impairment, 96 (27%) experienced neurological sequelae, and 29 (7.6%) died. Baseline factors associated with an ACO on univariate analysis included seizures, abnormal fontanelle, abnormal developmental status, CSF protein > 120 mg/dl, CSF glucose < 20 g/dl, abnormal neurological exam, mechanical ventilation and absence of fever (P< 0.05). On logistic regression analysis, abnormal neurological exam (OR, 6.54; 95% CI, 1.97-21.71; P=0.002), abnormal fontanelle (OR, 2.29; 95% CI 1.19-4.43; P=0.013) and mechanical ventilation (OR, 5.56; 95% CI, 1.87-16.51; P=0.002) were independent predictors of an ACO and were validated internally with bootstrapping.

**Conclusion:**

ACO occurred in 44% children with CABM; independent predictors included abnormal neurologic exam, abnormal fontanelle and mechanical ventilation.

**Disclosures:**

Rodrigo Hasbun, MD MPH FIDSA, Biomeriaux: Grant/Research Support|Biomeriaux: research funding and personal fees to help with Monte Carlo simulation studies evaluating impact of cost on adults and children

